# FPGA-Enabled, Smart Implants: A Path to Personalized Healthcare

**DOI:** 10.1109/OJEMB.2026.3720369

**Published:** 2026-08-04

**Authors:** Christos Strydis, David Veselka, Job van der Kleij, Muhammad Ali Siddiqi

**Affiliations:** Neuroscience DepartmentErasmus Medical Center 3015 GD Rotterdam The Netherlands; Quantum & Computer Engineering DepartmentDelft University of Technology 2628 CD Delft The Netherlands; Quantum and Computer Engineering DepartmentDelft University of Technology 2628 CD Delft The Netherlands; Electrical Engineering DepartmentLahore University of Management Sciences 66687 Lahore 54792 Pakistan; Quantum and Computer Engineering DepartmentDelft University of Technology 2628 CD Delft The Netherlands; Neuroscience DepartmentErasmus Medical Center 3015 GD Rotterdam The Netherlands

**Keywords:** Energy efficiency, embedded AI, field-programmable gate array, heterogeneous system, implantable medical device

## Abstract

*Goal:* Contrary to the conventional view that Field-Programmable Gate Arrays (FPGAs) are poorly suited to the stringent energy and resource constraints of Implantable Medical Devices (IMDs), this work investigates their potential to improve computational performance and energy efficiency in this domain. We propose heterogeneous CPU–FPGA systems as a means to support the growing demand for embedded AI in next-generation IMDs for intelligent and personalized healthcare. *Methods:* We evaluated heterogeneous CPU–FPGA architectures against CPU-only configurations across representative AI-driven neural and security IMD workloads. *Results:* FPGAs achieved lower execution latency and improved energy efficiency than CPU-based implementations in computationally intensive neural workloads, despite higher instantaneous power consumption in some cases due to parallel execution. In low-duty-cycle security workloads where aggressive power gating is feasible, FPGA- and CPU-based solutions exhibited comparable overall performance. *Conclusions:* These findings demonstrate that heterogeneous CPU–FPGA systems can integrate advanced AI and security algorithms into IMDs while satisfying battery-life, power-density, real-time processing, and practical implantability constraints relevant to long-term medical-device deployment, enabling intelligent, personalized, and secure implantable healthcare.

## I. Introduction

Implantable Medical Devices (IMDs) are rapidly evolving from simple, low-power systems into intelligent platforms capable of executing emerging artificial intelligence (AI)/machine learning (ML)-oriented processing workloads for advanced neural interventions [1], [2], [3]. Recent advances in AI have enabled real-time interpretation of complex neural signals [4], [5], promising patient-specific improvements in neurotherapeutic outcomes. Yet, as implants increasingly communicate via mainstream wireless protocols such as Bluetooth LE, traditional IMD designs, optimized for static, low-power operation, are facing unprecedented demands in both computing and security [3], [6].

This paradigm shift introduces two key challenges. First, emerging AI/ML-oriented functionalities raise computational demands inside implants, requiring architectures that execute complex algorithms with low latency and support in vivo updates, from security patches to adaptive control. Second, wireless connectivity exposes implants to privacy and cybersecurity risks that conventional designs cannot effectively mitigate [6], [7]. These constraints also introduce broader safety, reliability, and long-term deployment considerations relevant to practical medical-device regulation and implantability.

We propose addressing these challenges through the integration of reconfigurable-fabric logic, namely tiny Field-Programmable Gate Arrays (FPGAs), within future IMDs (Fig. 1). Despite skepticism regarding their suitability for ultra-low-power environments, FPGAs offer real-time, energy-efficient computation and post-deployment reconfigurability, transforming implants from static medical tools into adaptive, secure computing platforms. However, questions remain regarding their energy efficiency, latency impact, and compliance with stringent medical regulations.

**Fig. 1.**
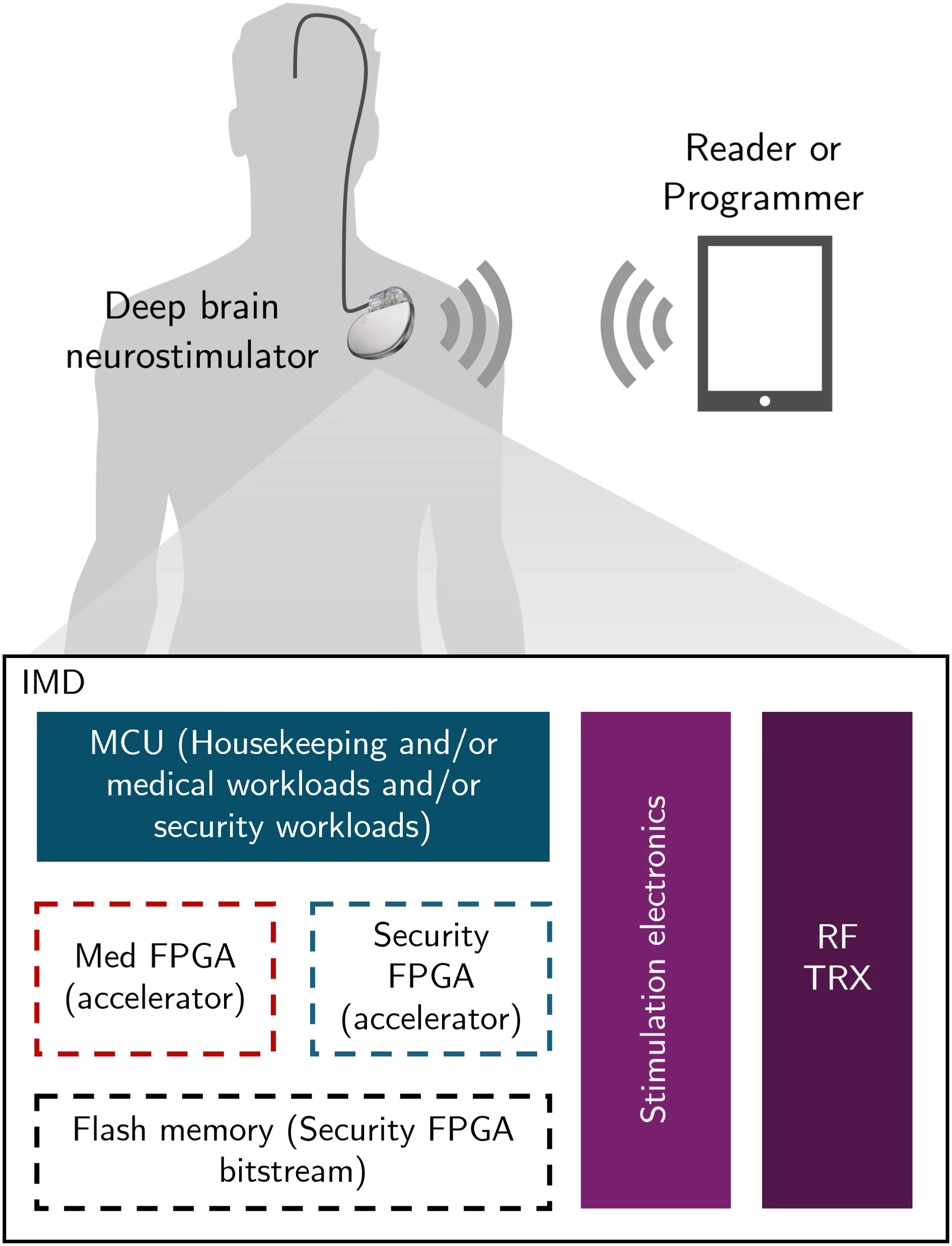
*Top:* An example IMD (deep-brain neurostimulator). *Bottom:* Major energy consumers in a typical IMD. The dotted boxes indicate the blocks added to study FPGA feasibility.

Early explorations of reconfigurable logic in implants [8], [9] demonstrated proof-of-concept designs but did not reach clinical translation. Instead, modern implants predominantly employ Microcontroller Units (MCUs) or CPUs, sometimes augmented with dedicated ASIC blocks for cryptographic tasks (e.g., AES engines) [10]. While ASICs offer excellent power efficiency, their rigidity limits adaptability to evolving medical and security requirements. Besides, GPUs, though highly parallel, are often too power-hungry for IMDs. In contrast, FPGAs, though typically more power-hungry, can be reprogrammed post-implantation, support algorithmic evolution, and remain cost-effective for low-to-medium device volumes.

Recent research has begun to re-evaluate FPGAs’ role in energy-constrained biomedical systems. Khan and da Silva [11] review FPGA applications in wearable devices, identifying low-power families and techniques such as sleep modes, voltage scaling, partial reconfiguration, and hybrid flash architectures: approaches directly relevant to IMDs. Similarly, Vaithianathan et al. [12] outline strategies for optimizing FPGAs for implantable and wearable devices, discussing components and design principles to enhance efficiency, though without empirical validation. Altman et al. [13] provide a broader review of FPGA applications in medical machine learning, highlighting the growing role of reconfigurable hardware in biomedical computation.

Despite these developments, FPGA-enabled implants remain underexplored experimentally. In particular, a systematic assessment jointly considering AI-driven neural workloads, security processing, physiological duty-cycle constraints, FPGA reconfiguration overheads, battery-life implications, and regulatory considerations in the context of next-generation secure and intelligent implants is still missing. In this work, we examine whether FPGAs can realistically serve as viable building blocks for such systems by evaluating their performance in energy-constrained conditions and analyzing their implications for future regulatory and design frameworks. Our findings aim to chart a path toward adaptive, updatable, and secure medical implants that bridge the gap between computational neuroscience, embedded systems, and cybersecurity. Concisely, the contributions of this work from a system-level feasibility and deployment perspective are as follows:

1) A systematic comparison of MCU-only and heterogeneous MCU–FPGA IMD architectures across representative neural, cardiac, and security workloads under realistic implant constraints.
2) An analysis of the interaction between physiological duty cycles, FPGA power-gating opportunities, and SRAM-based FPGA reconfiguration overheads in implantable systems.
3) A realistic assessment of battery-life, power-density, and implantability implications across representative IMD operating scenarios.
4) A discussion of deployment and regulatory implications of reconfigurable logic in IMDs under existing medical-device frameworks.

## II. Materials and Methods

The feasibility of employing FPGAs as accelerators in future implants depends critically on the design assumptions and the representativeness of the evaluated workloads. This section outlines the adopted methodology, study parameters, and boundary conditions, together with the selection criteria for processing devices, benchmarks, and workloads. It also details the measurement setup and performance metrics used, establishing a consistent and reproducible framework for evaluation.

### A. Boundary Conditions

To ensure a comprehensive evaluation and maximize the experimental realism, we selected two contrasting use cases: a minimal-workload cardiac IMD (e.g., a pacemaker) and a computationally intensive neural IMD (neurostimulator) employing a neural-network-based algorithm. Furthermore, neurostimulators face inherently greater demands due to the stringent real-time constraints imposed by rapidly changing neural signals, in contrast to the comparatively slower cardiac signals processed by pacemakers. Fig. 2 demonstrates that the average inter-spike interval of a Purkinje-cell signal in the cerebellum is orders of magnitude shorter than that of a cardiac signal.

**Fig. 2.**
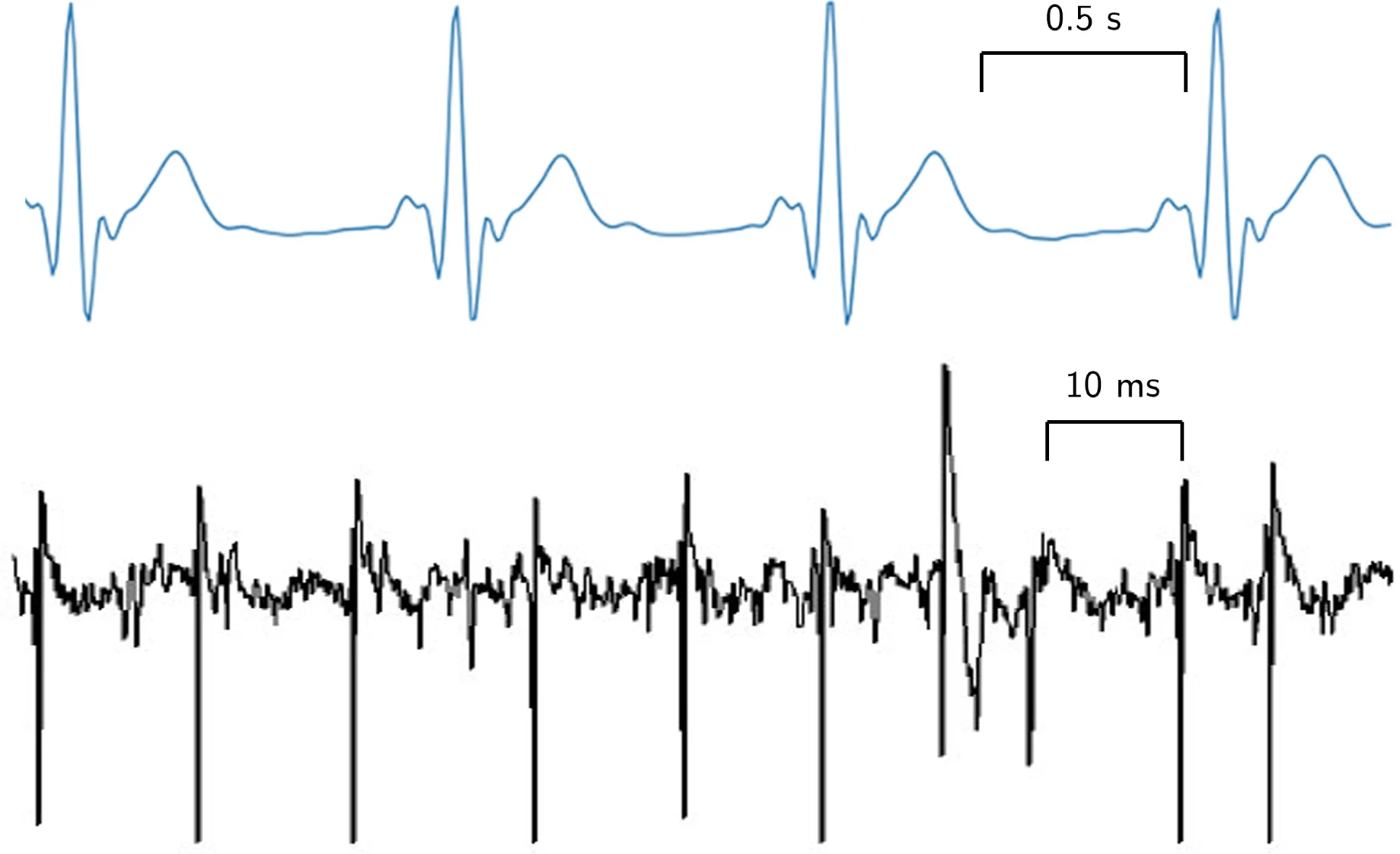
Physiological signals and the associated time scales. *Top:* A typical cardiac signal. *Bottom:* A (single-unit) neural signal recorded from the cerebellum.

### B. Device Choice

The choice of processing devices is critical for evaluating next-generation IMD workloads. In this study, we focus on heterogeneous MCU–FPGA architectures, comparing conventional MCU-based implementations against FPGA-accelerated solutions for representative medical and security workloads.

#### 1) CPU

MCUs remain the baseline processing units for IMDs due to their integration of CPUs, memory, and peripherals. For this study, the Tiny Gecko 11 MCU (EFM32TG11) from Silicon Labs was selected that has the Cortex-M0+ processor from ARM [10]. This device is optimized for low-power applications and includes dedicated hardware for AES encryption, providing a useful comparison for FPGA implementations.

#### 2) FPGA

The Lattice iCE40UP5K was selected as the primary FPGA [14] due to its low-power characteristics and reconfigurability, critical for IMD use cases (see Table I). The reported frequency range corresponds to the operating conditions employed in the experiments and should not be interpreted as the architectural maximum frequency capability of the FPGA fabric.

**TABLE I:** Device Characteristics

Device Name	Freq. range used in study (MHz)	Voltage (V)	Current-Measurement Method
Tiny Gecko 11	0.001–48	1.8–3.3	Advance Energy Monitoring via IDE
iCE40UP5K	0.01–48	1.2	Manually via test points 11 & 12 (VCC)

### C. Assumptions and Constraints

To ensure consistency, several realistic assumptions were made:

• *Data-Transfer Rate and Volume:* A transfer rate of 265 kbps was assumed, reflecting the maximum rate employed by a commercial implantable-grade transceiver [15]. Best-case and worst-case transfer durations were calculated for data volumes ranging from 1 MB *monthly* transmissions to 10 MB *daily* transmissions. This range encompasses typical patient accesses using their smartphones and more detailed data transfers associated with readers/programmers of specialists.
• *Duty Cycling:* IMDs are assumed to operate in cycles of active and sleep modes to conserve energy. Each workload is modeled as a periodic task consisting of an active execution interval followed by an idle interval. Duty cycle is defined as the ratio between active execution time and total workload period. The duty cycling percentages are dependent on the workload type, implant type (cardiac or neural), and the data-transfer volume (discussed above). This analysis considers both clock gating (CG) and power gating (PG) as FPGA sleep mode strategies. For the EFM32TG11, ‘power gating’ refers to the deepest low-power sleep mode employed in this study. For the iCE40UP, wake-up from PG requires full SRAM configuration reload from external SPI flash memory, introducing millisecond-scale reconfiguration latency.
• *Reconfiguration Costs:* The analysis accounted for the energy and time overhead of FPGA reconfiguration, particularly during wake-up from power-gated sleep states. While the iCE40UP5K includes both SRAM and Non-volatile Configuration Memory (NVCM) for the active configuration (bitstream), the *one-time* programmability of NVCM necessitates the use of an external SPI Flash (MX25V1006F [16]) for non-volatile bitstream storage. Consequently, FPGA wake-up from PG requires full bitstream reload from external flash memory.
• *Environmental Context:* Section II-A highlights the disparity in computational complexity between cardiac and neural environments, with the latter demanding significantly greater processing power. This difference extends to stimulation strategies, where the unique patterns required for neural modulation result in differing stimulation current consumption compared to cardiac pacing (see Table II). The parameters summarized in the table were used as system-level assumptions for workload scheduling, duty-cycle estimation, stimulation activity, communication activity, and battery-life analysis throughout the evaluated scenarios. These operating assumptions were derived from published literature, commercial-device specifications, and prior implant-research experience [7].

### D. Workloads

To evaluate the devices, a series of workloads and scenarios were defined. These workloads encompass both medical and security algorithms, designed to represent real-world use cases for IMDs. An *iteration* refers to one complete execution of the target workload for a single processing event or processing window, depending on the workload type. Two categories of workloads were employed:

#### 1) Medical Algorithms

Next-generation neural workloads, exemplified by spike sorting [17] and Current-Source Density (CSD) [18] algorithms, present significant computational challenges. Spike sorting, encompassing spike *detection* and *classification*, was chosen for its significant computational demands and direct applicability to neural IMDs. We selected spike sorting of Purkinje-cell neural signals (single-unit recordings) from the cerebellum, a process particularly relevant for next-generation implants designed to address brain injuries and motor function deficits. Furthermore, spike classification, performed using a multilayer perceptron (MLP), serves as a representative, embedded AI/ML workload with nontrivial computational demands and limited gating opportunities.

CSD [18], a less computationally intensive algorithm, was included for comparative analysis due to its utility in detecting events within Local-Field Potential (LFP) signals, rather than the single-unit signals previously discussed. Similar to single-unit signals, LFP signals are a critical data type recorded by neural IMDs.

Additionally, spike detection [19] was employed to represent simpler cardiac workloads. In this context, it is used to detect heartbeats, allowing for heart rate computation (using a basic counter) for pacing management.

#### 2) Security Algorithms

Given the critical function of IMDs and the sensitive patient data they handle, robust security primitives are essential. To benchmark security algorithms, we implemented AES-128 (block cipher) to serve as the mainstream primitive, alongside lightweight alternatives such as SIMON/SPECK (block cipher) and PHOTON (hash function), to evaluate energy-efficient cryptographic workloads. Given that numerous modern MCUs, such as the EFM32TG11, incorporate cryptographic accelerators for AES-128, we also included hardware AES (HW AES) measurements from the EFM32TG11 for comparative analysis.

The disparate duty-cycling demands of medical and security workloads, coupled with their independent nature and the iCE40UP’s inherent limitations regarding partial reconfiguration, necessitate the use of two FPGAs, each dedicated to its respective workload, as illustrated in Fig. 1.

### E. Scenarios and Processing-Device Configurations

Four scenarios were developed to evaluate the devices under varying conditions:

1) *Cardiac* IMD with a data-transfer volume of 1-10 MB per *day*.
2) *Cardiac* IMD with a data-transfer volume of 1-10 MB per *month*.
3) *Neural* IMD with a data-transfer volume of 1-10 MB per *day*.
4) *Neural* IMD with a data-transfer volume of 1-10 MB per *month*.

The following processing-device configurations were evaluated across the defined scenarios:

1) *MCU only:* Both the medical and security workloads are implemented as software on EFM32TG11, which enters power gating during idle periods. Due to the stringent real-time constraints of neural signals, power gating is limited to cardiac spike detection and security workloads, and cannot be applied to neural spike sorting.
2) *MCU only (HW AES):* Same as above except that it utilizes EFM32TG11’s hardware-accelerated AES-128, rather than a software implementation.
3) *MCU (Med) + FPGA (AES):* This is a hybrid solution in which medical workloads are executed on the MCU and security workloads on the FPGA. Power gating is applied to both the devices (but excluding neural spike sorting, as discussed above).
4) *FPGA only:* Both the medical and security workloads are executed on the respective FPGAs (see Fig. 1).
5) *FPGA only (CG):* Same as *FPGA only* but with clock gating for power savings during inactivity. Because of the strict real-time requirements of neural signals, clock gating is only feasible for cardiac spike detection and security, not for neural spike sorting.
6) *FPGA only (PG):* Same as *FPGA only (CG)* but with power gating during communication/security inactivity. While cardiac signal timing enables clock gating on the FPGA, power gating remains impractical due to the resulting millisecond-scale bitstream reconfiguration delays. Due to iCE40UP’s SRAM-based architecture, an external flash memory is required for bitstream storage.

Fig. 3 summarizes the workloads and scenarios mentioned above. Table II describes the specifications of the assumed cardiac and neural IMDs.

**Fig. 3.**
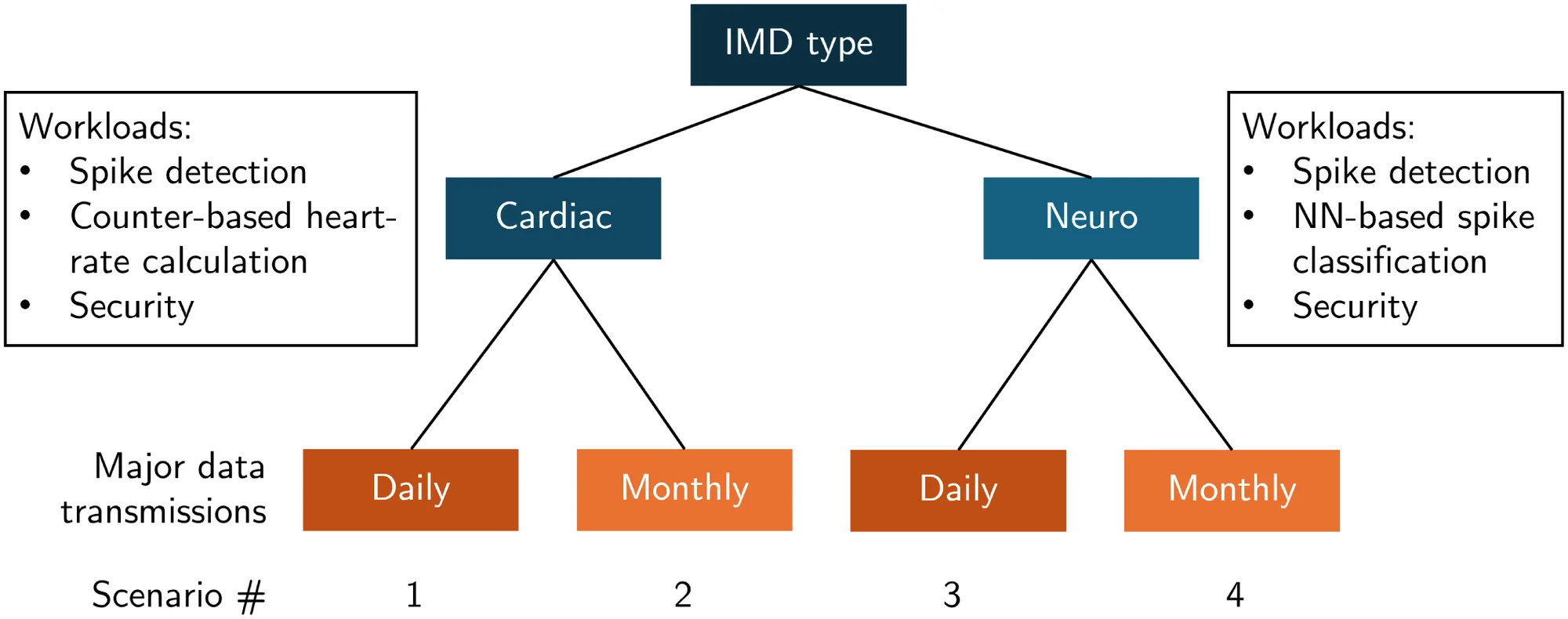
Scenarios assumed in this study based on IMD/workload type and the volume of wireless data transmissions.

### F. Measurements

Current and workload execution time were measured using distinct methods for each device (see Table I).

#### 1) CPU/MCU

For the EFM32TG11, live measurements were performed utilizing the advanced energy monitoring (AEM) feature of the SLSTK3301A starter kit and the Simplicity Studio IDE. The AEM infrastructure provides subsystem-level current monitoring of the EFM32TG11 device rather than total development-board consumption. Unused peripherals and onboard subsystems were disabled where possible during measurements. Additionally, one of the internal timers was employed to determine the run times of individual workloads.

**TABLE II:** Pacemaker and Neurostimulator Specifications

**Parameter**	**Value**
Supply voltage	1.8 V (MCU), 1.2 V (FPGA)
Clock frequency	12 MHz
RF-transceiver effective data rate	265 kbps (maximum value) [15]
Battery capacity	6.3 Ah [20]
**Pacemaker:**	
Stimulation energy	20 *μ*J per pacing pulse [21]
Average no. of pacing events / day	10000 [22]
Sampling rate	1000 Hz [23]
**Neurostimulator:**	
Stimulation current	12 mA [24]
Pulse width	1 ms [24]
Pulse freq.	333 Hz [24]
Burst duration	10 seconds [24]
Average seizures / day	4.3 [25]
Sampling rate	24414 Hz [17]

#### 2) FPGA

The iCE40 UltraPlus Breakout Board, which houses the iCE40UP5K FPGA, provides test points (TPs) connected to specific voltage lines for measuring current consumption via multimeters or oscilloscopes. Measurements focused on the FPGA-related supply rails (VCC) exposed through these test points rather than total board-level consumption. Unused peripherals and non-essential subsystems were assumed inactive during the experiments. The workload run times were calculated using RTL simulation. All FPGA implementations successfully met timing closure at 12 MHz under the standard iCE40UP5K synthesis and place-and-route flow; Table III summarizes the achieved resource utilization for each workload.

**TABLE III:** FPGA Resource Utilization (iCE40UP5K)

**Workload**	**LUTs**	**FFs**	**BRAM**	**DSP**	**I/O Buffers**
Spike detector	249	124	0	5	14
Spike classifier	3184	1125	2	8	3
CSD	1034	619	0	2	11
AES-128	2026	833	0	0	13
SIMON/SPECK	305	214	0	0	12
PHOTON	276	188	0	0	12

Because the MCU and FPGA measurements were performed on different development platforms, the reported results should be interpreted as processor-focused rather than complete board-level power comparisons. Whereas direct measurements were used for both MCU and FPGA implementations, eFPGA values were obtained from vendor-provided models as discussed in Section III-C.

### G. Evaluation Metrics

A comparative analysis of CPU and FPGA implementations was conducted, examining **execution time**, **average power** consumption per workload iteration, **energy consumption** per iteration, and **power density**. Execution time, average power, and energy consumption are key metrics for evaluating real-time behavior and impact on device autonomy. Power density is crucial for determining the thermal profile and safety of IMDs implanted adjacent to human tissue, specifically regarding the risk of localized hot spot development. It should remain below the reported limit for clinical grade implants of approximately 62 mW/cm$^{2}$ [26].

Furthermore, a comprehensive assessment of **battery life** was conducted by comparing expected operational durations across diverse scenarios and CPU/FPGA configurations, taking into account workload demands (derived from physiological signal processing) and communication volume. Execution-time and power measurements were obtained directly from the evaluated workloads and platforms, whereas the parameters listed in Table II were incorporated into the higher-level battery-life and scenario analysis. Battery life was estimated from the aggregate energy consumption of active workload execution, idle/sleep intervals, wireless communication, and stimulation activity: $E_{\mathrm{total}} = E_{\mathrm{workload}} + E_{\mathrm{idle}} + E_{\mathrm{comm}} + E_{\mathrm{stim}}$.

## III. Results

### A. Performance Analysis: CPU/MCU Vs. FPGA

Using the platform parameters outlined in Table II, Fig. 4 presents a comparison of iCE40UP and EFM32TG11 across the medical and security workloads selected in Section II-D. In the figure, the reported execution time per iteration corresponds only to the active workload execution interval and excludes idle/sleep periods between successive workload activations.

**Fig. 4.**
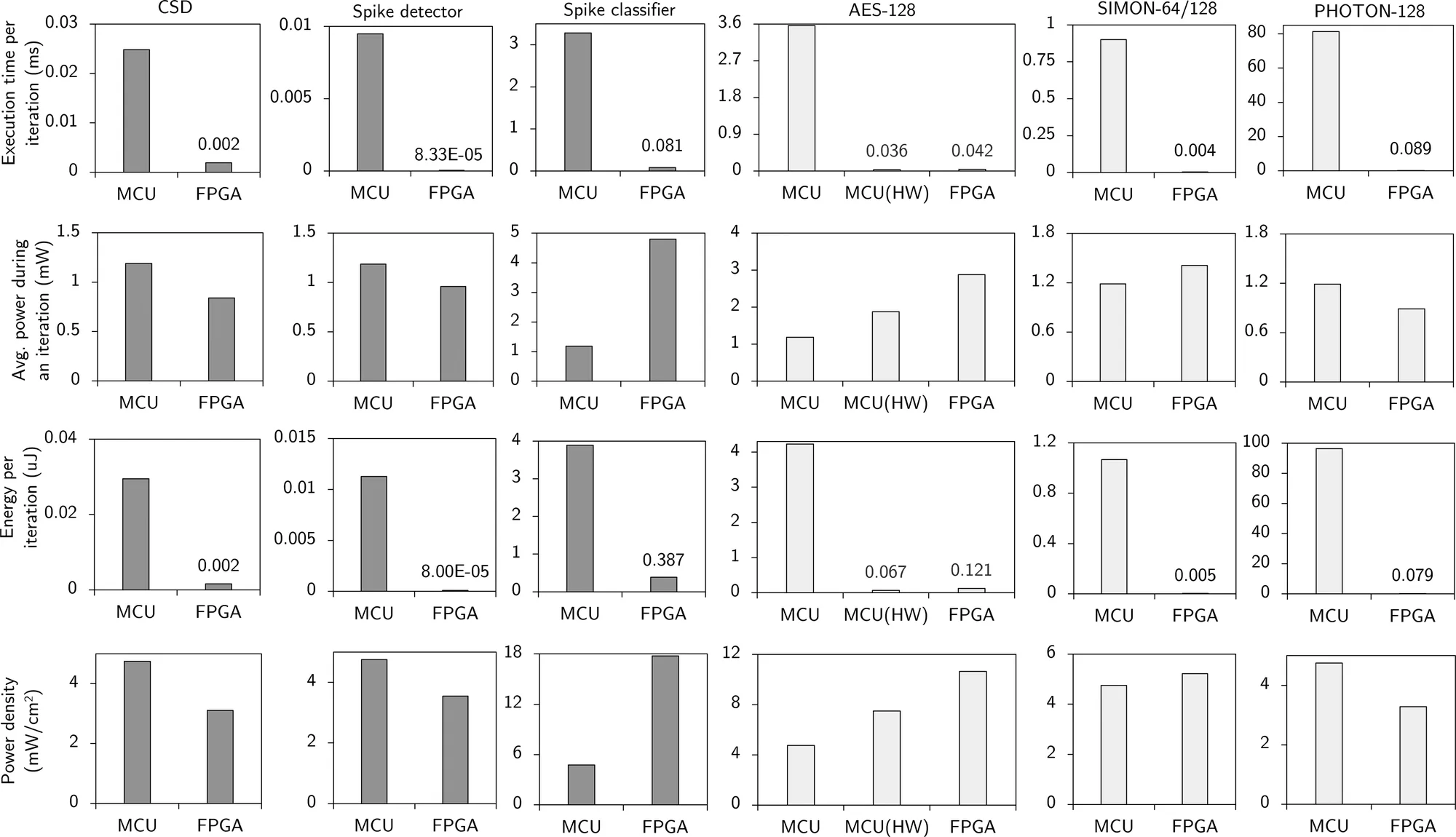
Performance comparison of EFM32TG11 (MCU) and iCE40UP (FPGA) across medical and security workloads. MCU(HW) denotes hardware-accelerated AES on the EFM32TG11. The FPGA achieves lower execution times for most workloads due to parallel execution. Although instantaneous power may increase in some cases, the reduced execution time often results in improved overall energy efficiency.

As anticipated, the iCE40UP demonstrates superior execution times compared to the EFM32TG11 across all workloads, attributed to its inherent parallelism. The sole exception is the hardware-accelerated AES-128 implementation within the EFM32TG11’s cryptographic module, which outperforms the FPGA, as expected.

However, the average power consumption per workload iteration exhibits a more nuanced trend. For computationally intensive tasks, such as the MLP-based spike classifier and AES-128, increased parallelism in the FPGA results in a corresponding rise in power consumption.

Despite this power trade-off, the shorter execution times achieved by the FPGA significantly reduce overall energy consumption, leading to superior energy efficiency compared to the CPU-based implementations.

Power density trends closely align with power consumption, given the comparable die areas of the EFM32TG11 and iCE40UP. Notably, both devices remain below the prescribed power density limit of 62 mW/cm$^{2}$ (discussed in Section II-G).

### B. Contextual Analysis: Battery Life & IMD Usage Scenarios

The results presented thus far suggest that FPGAs offer a performance advantage in IMD applications for many workloads. However, to comprehensively evaluate system-level implications, further investigation is required, specifically concerning: (1) the optimization of MCU–FPGA pairings, (2) the influence of realistic usage patterns in cardiac and neural settings, and (3) the sensitivity to data transmission volume. Fig. 5 illustrates the battery life characteristics of different IMD processing-device configurations under the four scenarios defined in Section II-E. The battery-life analysis accounts for workload execution energy, idle/sleep energy, communication energy, and stimulation energy using the assumptions summarized in Table II. Consequently, the observed battery-life improvements depend not only on gating opportunities, but also on workload execution efficiency and workload duty-cycle characteristics.

**Fig. 5.**
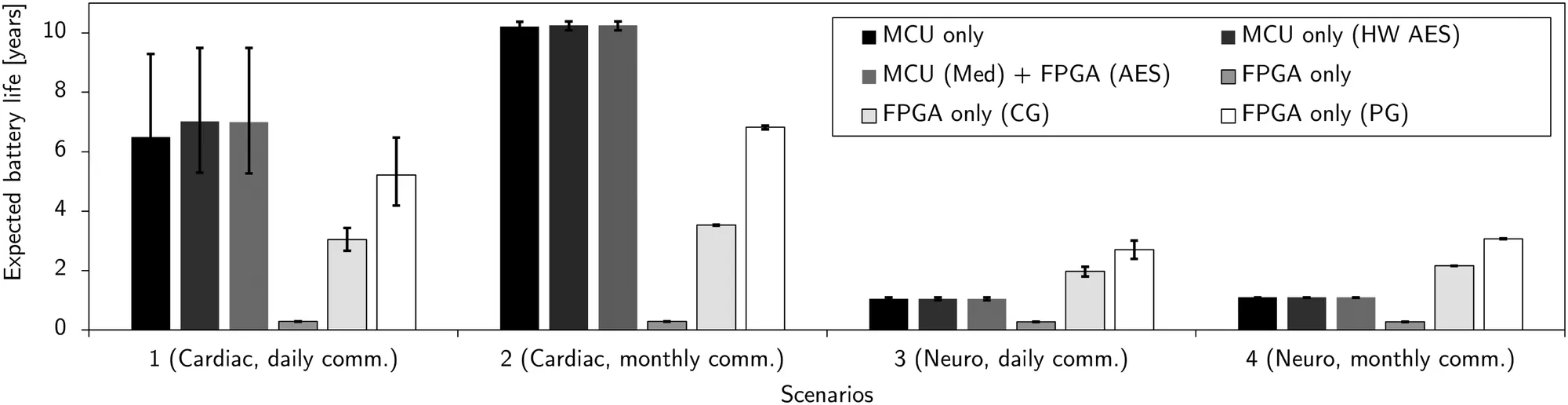
Battery life of IMD processing-unit configurations across the four evaluated scenarios. Bars represent the nominal transmission case (5-MB daily for scenarios 1 and 3; weekly for scenarios 2 and 4), while whiskers indicate sensitivity to communication volume, spanning 10 MB (lower limit) and 1 MB (upper limit). Scenario definitions and processing-device configurations are provided in Section II-E. FPGA-based solutions improve battery life in neural workloads, whereas MCU-only solutions remain favorable in many cardiac scenarios due to limited FPGA power-gating opportunities.

The analysis reveals that FPGA (CG/PG) configurations offer enhanced battery life compared to MCU-only configurations for neural workloads. However, this trend reverses for cardiac workloads, where MCUs exhibit superior performance. Specifically, FPGAs contribute to extended battery life in high-duty-cycle and highly-parallel neural applications, which offer limited gating opportunities. In these workloads, the primary benefit arises from reduced active execution time and lower energy consumption per workload iteration, rather than aggressive power gating. Although FPGA power gating opportunities remain limited in neural workloads, the reduced execution time substantially decreases the active energy contribution to the overall battery budget. Conversely, MCUs demonstrate better performance in lower-duty-cycle cardiac workloads, primarily because the cardiac duty cycles are not low enough for FPGAs to employ power gating. However, when considering very-low duty cycle security workloads that *do* permit FPGA power gating, the performance of FPGAs and MCU-only implementations was comparable.

### C. The Use of eFPGAs

In addition to neural workloads, the above analysis confirms the suitability of FPGAs for security workload implementation in modern IMDs. As a *complementary* investigation, we introduced an eFPGA-based security workload implementation for comparison. We used Menta’s eFPGA Core IP [27], which was synthesized in TSMC 28 nm technology. Power consumption was estimated using Menta’s Origami Designer and provided models, since physical current measurements were not possible due to the lack of a fabricated device. The eFPGA clock frequency and supply voltage were set to 12 MHz and 1.2 V, respectively, matching the iCE40UP.

Fig. 6 illustrates the performance comparison between the eFPGA/MCU hybrid (security-focused eFPGA), MCU-only, and MCU/iCE40UP solutions. While the eFPGA was expected to exhibit lower power consumption, its power usage is comparable to the iCE40UP, which is attributed to the considerable leakage current inherent in the 28 nm process.

**Fig. 6.**
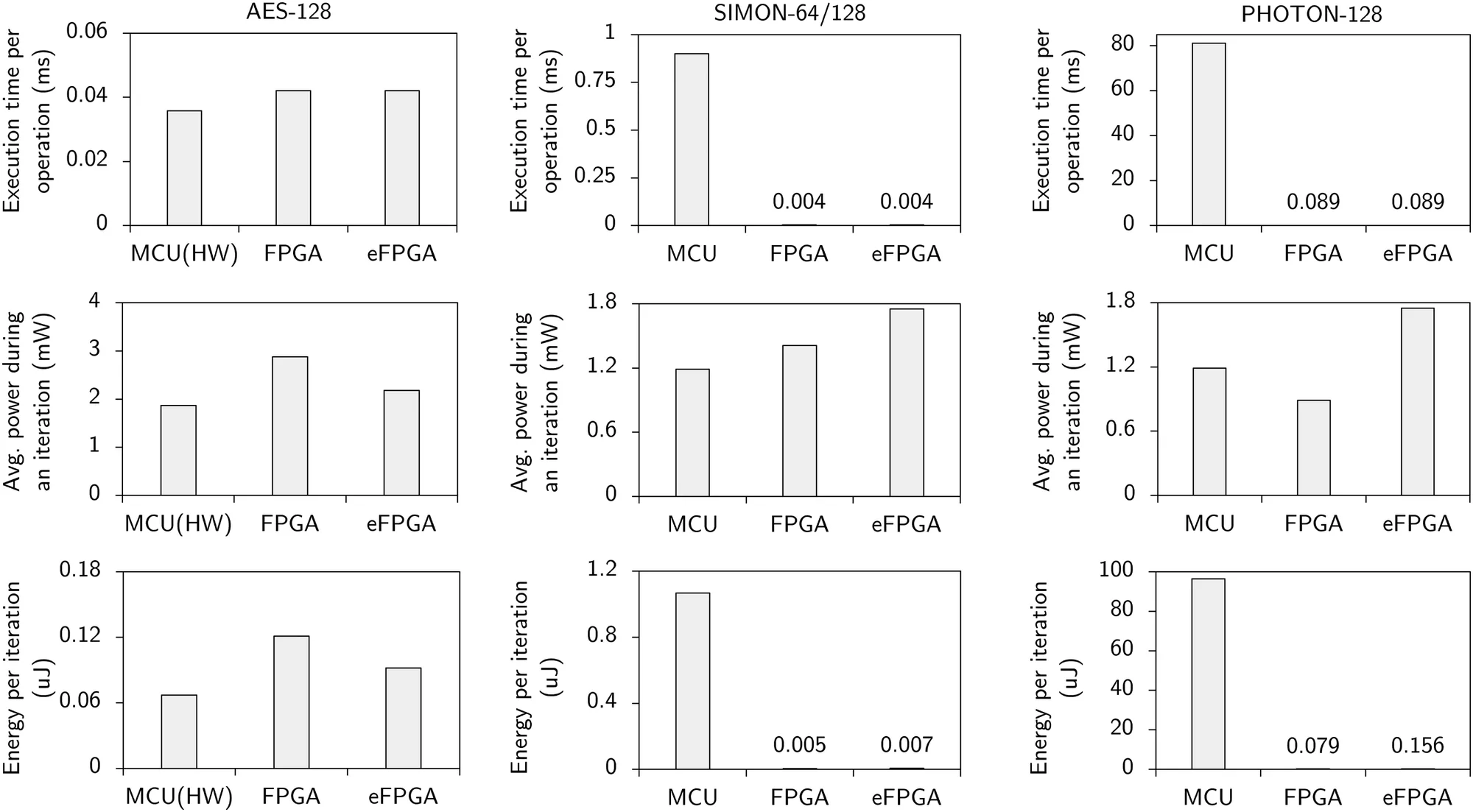
Performance comparison of eFPGA with EFM32TG11 (MCU) and iCE40UP (FPGA) across security workloads. The evaluated eFPGA implementation exhibits execution-time and power characteristics comparable to the standalone FPGA implementation.

Due to the similar power consumption and identical workload execution times observed for the eFPGA and iCE40UP, the resulting battery life impact is insignificant, as shown in Fig. 7.

**Fig. 7.**
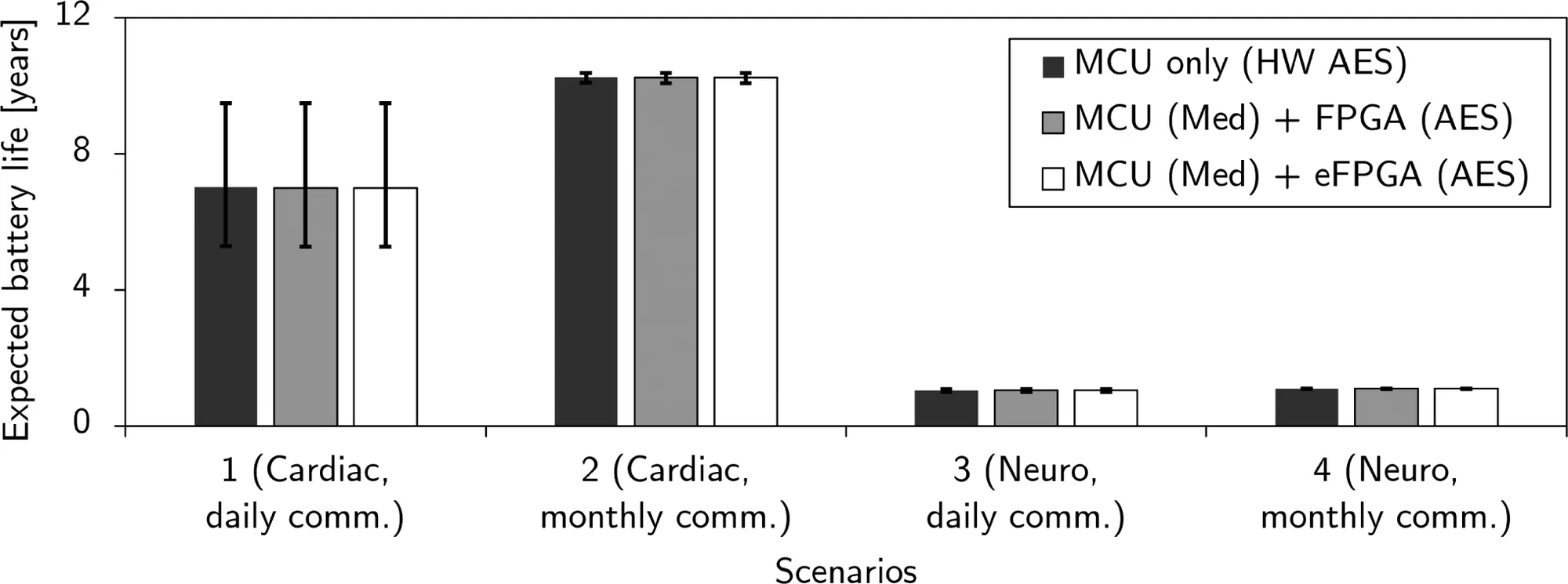
Impact of eFPGA on battery life. Because the evaluated eFPGA implementation demonstrates similar energy characteristics to the standalone FPGA, the resulting battery-life differences remain modest across the considered scenarios.

## IV. Discussion

### A. Regulatory Considerations

Our feasibility study would be incomplete without a thorough examination of the regulatory implications of integrating FPGAs into active IMDs. These devices fall under strict oversight by agencies such as the U.S. Food and Drug Administration (FDA) and the European Medicines Agency (EMA), which require proof of safety, efficacy, and functional reliability. Any modification affecting a device’s performance, whether hardware or software, typically mandates regulatory review.

IMDs such as pacemakers and neurostimulators are classified as *Class-III* devices under *MDR2017/745* due to their invasive, life-critical nature [28]. Certification involves rigorous evaluation by Notified Bodies in the EU [29] or by the FDA in the USA. FPGAs introduce flexibility via reconfigurable logic, enabling real-time optimizations post-implantation, yet regulatory authorities treat any functional change as a potential trigger for re-evaluation [30]. Key determinants include: (1) the extent of functional change, (2) its impact on patient safety, treatment efficacy, and device reliability, and (3) the validation and verification processes assessing FPGA reconfiguration.

#### 1) In-Vivo Reconfiguration Implications

The regulatory response to in vivo FPGA reconfiguration depends on the modification’s scope. Major changes that alter core functionality or introduce risk require review, whereas pre-approved updates within defined safety boundaries may proceed without full recertification [31]. Notably, **regulators treat FPGA configurations analogously to software**, meaning that their reconfigurability does not automatically trigger additional certification hurdles [32], [33]; emphasis lies on verification of function rather than implementation details. FPGAs are generally regarded as “black boxes” unless they perform safety-critical operations [32]. Factors influencing approval include:

• Whether FPGA modifications remain within an approved operational envelope.
• The device’s ability to maintain consistent, validated performance across configurations.
• The regulatory classification of the IMD and degree of control over in-field updates.

#### 2) Recertification Triggers and Regulatory Pathways

Recertification requirements depend on the update magnitude: **Minor updates** (e.g., bug fixes or algorithm refinements) may require documentation, *notification* or limited review depending on the applicable regulatory pathway [34]. **Major updates** that affect safety or efficacy may trigger *re-evaluation* within existing certification cycles, typically every five years under *MDR2017/745, Annex VII, Section 4.11* [28].

Regulatory frameworks offer established pathways for managing such updates: The FDA allows Premarket Approval (PMA) supplements or Premarket Notification (510(k)) submissions for significant changes [35], while preapproved update protocols may reduce or avoid the need for complete recertification if FPGA changes remain within verified design boundaries [31]. Risk-based assessments under *ISO 14971* guide whether reconfiguration warrants additional filings [36].

#### 3) Applicable Standards and Verdict

Finally, *expert commentary and industry sources* suggest that FPGA updates follow the same rules as software updates, without additional obligations compared to MCU-based systems [32], [34], [37], [38]. This is confirmed in the *IEC 62304* standard, governing software life-cycle processes, which implicitly covers FPGA code as a form of complex design unless the design qualifies as pure (e.g., easy-to-test) hardware under *IEC 60601-1* [33], [39]. Its 2015 update formalized FPGAs within the software life-cycle framework, closing any potential regulatory loopholes.

### B. Application Suitability: Cardiac Vs. Neural IMDs

The analysis suggests that FPGAs may not be optimal for cardiac-IMD medical functions, primarily because the duty cycles enforced by the physiological deadlines are not low enough to allow FPGA power gating, unlike MCU implementations. However, FPGAs demonstrate significant potential for neurostimulators. This is particularly advantageous given the parallel and computationally intensive algorithms employed in neurostimulation, which must process rapidly changing and often noisy neural signals.

### C. Extended Battery Life in Neurostimulators

By leveraging the power efficiency of FPGAs for neural workloads, it is feasible to design neurostimulators that are either non-rechargeable or require significantly less frequent recharging. This extended operational lifespan is crucial for patient comfort and reduced maintenance.

### D. Enhanced Security and Upgradability

Even when the medical algorithm is implemented on an MCU, FPGAs offer a compelling solution to implement robust security schemes. This is especially relevant because security algorithms often require frequent updates to address evolving threats. Accelerating these security workloads, particularly with power gating, proves to be highly effective.

Besides, emerging ultra-low-power AI/ML accelerators may offer improved efficiency for specific inference tasks, compared to FPGAs. However, such architectures are often optimized for narrow workload classes and may not provide the flexibility required to support the broader mix of medical, security, and adaptive workloads targeted in this study.

### E. Limitations of Power Gating in Medical Workloads

The inherent constraints imposed by the real-time requirements of physiological signals, specifically in medical workloads, preclude the effective use of power gating for algorithm acceleration. The timing demands of these signals do not allow sufficient time for SRAM-based FPGA reconfiguration from external flash, rendering power gating impractical for neural and many cardiac workloads.

### F. Power-Density Compliance

The power density observed in our FPGA-based IMD implementations remains well below the clinically prescribed upper limit of approximately 62 mW/cm$^{2}$ [26]. This ensures compatibility with stringent safety standards for implantable medical devices.

Table IV summarizes this analysis, which underscores the suitability of FPGAs for compute-intensive IMD workloads characterized by high duty cycles. Furthermore, FPGAs demonstrate optimal performance in scenarios with extremely sparse usage, particularly security workloads, due to the applicability of power gating. Medium duty cycle applications, such as those in cardiac IMDs, could theoretically benefit from power gating; however, this is currently impractical due to the high reconfiguration latency of SRAM-based FPGAs. Future IMD designs incorporating flash-based FPGAs may overcome these limitations, enabling reconfiguration with significantly lower energy and performance overhead.

**TABLE IV:** Summary of Results

Workload Type	Duty Cycling	Deepest Sleep Mode		FPGA Feasibility
MCU	FPGA
Cardiac	Medium	PG	CG	Less Feasible
Neuro	High	N/A	N/A	Feasible
Security	Low	PG	PG	Feasible

### G. Study Limitations

The presented operating scenarios in this work should be interpreted as representative and exploratory rather than clinically predictive. Accordingly, the reported results should be viewed as directional for the evaluated design space, rather than universally predictive across all implant configurations, clinical use cases, or future low-power processing architectures. The analysis is intended to expose system-level architectural trends associated with alternative processing cores under consistent assumptions, rather than to provide a complete clinical deployment model for a specific implant product.

The presented conclusions are also derived from a limited set of evaluated platforms, namely a specific MCU, FPGA family, and eFPGA implementation operating under representative assumptions and workload scenarios. Consequently, the quantitative results should not be interpreted as universally generalizable across all future low-power reconfigurable architectures, process nodes, implant SoCs, or emerging heterogeneous accelerator platforms. Instead, they should be understood as comparative evidence of the architectural trade-offs observed within the evaluated design space.

Similarly, the battery-life estimates presented in this work should be interpreted as first-order, comparative projections rather than clinically predictive estimates. The model accounts for workload execution, idle/sleep behavior, wireless communication, and stimulation activity, but does not explicitly model advanced battery aspects, such as aging, temperature effects, long-term leakage evolution, and so on. These factors may affect absolute device lifetime and, in some regimes, stimulation energy may dominate computational energy. However, because stimulation and communication assumptions are held fixed across the processing configurations within each scenario, the reported comparisons primarily capture the relative contribution of the processing subsystem. The resulting trends should therefore be interpreted as directional architectural trade-offs under representative assumptions, rather than full electrochemical or clinical battery-life predictions.

## V. Conclusion

This work has demonstrated that FPGAs can serve as effective accelerators for IMDs, particularly for neural and security-intensive workloads. Our analysis indicates that while FPGAs may not be optimal for cardiac IMDs, mainly because the duty cycles dictated by physiological deadlines are insufficiently low to permit effective power gating, they exhibit significant potential for neurostimulation systems. This advantage arises from their ability to efficiently execute parallel, computation-intensive algorithms required to process rapidly fluctuating and often noisy neural signals. As a result, FPGA-based implementations can enhance device longevity by extending battery life and reducing recharge frequency, thereby improving patient comfort and minimizing maintenance requirements. Security remains a critical concern for IMDs, and FPGAs offer a robust platform for cryptographic acceleration. The results show that FPGAs can efficiently offload security tasks and maintain adaptability to evolving security threats through their reconfigurable nature. Furthermore, they comply with strict power-density and safety constraints essential for IMD applications. Future work will focus on exploring partial reconfiguration, flash-based FPGA technologies, hybrid FPGA-GPU architectures, and the deployment of more advanced AI models, including CNNs, spiking neural networks, adaptive online-learning pipelines, and larger neural-processing workloads, to further optimize performance and energy efficiency in next-generation IMDs.
